# RyRCa^2+^ Leak Limits Cardiac Ca^2+^ Window Current Overcoming the Tonic Effect of Calmodulin in Mice

**DOI:** 10.1371/journal.pone.0020863

**Published:** 2011-06-06

**Authors:** María Fernández-Velasco, Gema Ruiz-Hurtado, Angélica Rueda, Patricia Neco, Martha Mercado-Morales, Carmen Delgado, Carlo Napolitano, Silvia G. Priori, Sylvain Richard, Ana María Gómez, Jean-Pierre Benitah

**Affiliations:** 1 Inserm, U637, Université Montpellier-1, Université Montpellier-2, Montpellier, France; 2 Instituto de Investigaciones Biomédicas Alberto Sols CSIC-UAM, Madrid, Spain; 3 Inserm, U769, IFR141, Faculté de Pharmacie, Université Paris-Sud 11, Chatenay-Malabry, France; 4 Department of Biochemistry, CINVESTAV, Mexico City, Mexico; 5 Departamento de Farmacología, Facultad de Medicina, Universidad Complutense, CyB, CSIC, Madrid, Spain; 6 Molecular Cardiology, Fondazione Salvatore Maugeri, Pavia, Italy; 7 Cardiovascular Genetics, Leon Charney Division of Cardiology, Langone Medical Center, New York University School of Medicine, New York, United States of America; 8 Department of Cardiology, University of Pavia, Italy; 9 Inserm, U1046, Université Montpellier-1, Université Montpellier-2, Montpellier, France; Brigham and Women's Hospital - Harvard Medical School, United States of America

## Abstract

Ca^2+^ mediates the functional coupling between L-type Ca^2+^ channel (LTCC) and sarcoplasmic reticulum (SR) Ca^2+^ release channel (ryanodine receptor, RyR), participating in key pathophysiological processes. This crosstalk manifests as the orthograde Ca^2+^-induced Ca^2+^-release (CICR) mechanism triggered by Ca^2+^ influx, but also as the retrograde Ca^2+^-dependent inactivation (CDI) of LTCC, which depends on both Ca^2+^ permeating through the LTCC itself and on SR Ca^2+^ release through the RyR. This latter effect has been suggested to rely on local rather than global Ca^2+^ signaling, which might parallel the nanodomain control of CDI carried out through calmodulin (CaM). Analyzing the CICR in catecholaminergic polymorphic ventricular tachycardia (CPVT) mice as a model of RyR-generated Ca^2+^ leak, we evidence here that increased occurrence of the discrete local SR Ca^2+^ releases through the RyRs (Ca^2+^ sparks) causea depolarizing shift in activation and a hyperpolarizing shift inisochronic inactivation of cardiac LTCC current resulting in the reduction of window current. Both increasing fast [Ca^2+^]_i_ buffer capacity or depleting SR Ca^2+^ store blunted these changes, which could be reproduced in WT cells by RyRCa^2+^ leak induced with Ryanodol and CaM inhibition.Our results unveiled a new paradigm for CaM-dependent effect on LTCC gating and further the nanodomain Ca^2+^ control of LTCC, emphasizing the importance of spatio-temporal relationships between Ca^2+^ signals and CaM function.

## Introduction

Dynamic modulation of cellular Ca^2+^ flows from either the extracellular space or the intracellular Ca^2+^ store into the cytoplasm participates in key pathophysiological processes, which depends on the ability of cells to properly sort ‘global’ and ‘local’ Ca^2+^ signals [1]. In this respect, the functional coupling of the sarcolemmal L-type Ca^2+^ channels (LTCC) and the sarcoplasmic reticulum (SR) Ca^2+^ release channels (ryanodine receptor, RyR), plays an important role in ventricular cardiomyocytes [2], [3]. Depolarizing stimuli open voltage-gated LTCC, leading to Ca^2+^ entry (*I_Ca_*) and a subsequent rise in the cytoplasmic free Ca^2+^ concentration ([Ca^2+^]_i_). While such [Ca^2+^]_i_ elevations are initiated by LTCC, they are also influenced by Ca^2+^ transporting organelles such as the mitochondria and the SR. Notably, in response to these increases in [Ca^2+^]_i_, Ca^2+^ binds to and activates RyRs thereby amplifying the initial Ca^2+^ signal through the locally controlled Ca^2+^-induced Ca^2+^-release (CICR) process to support the excitation-contraction coupling (ECC) and thus heart function [4]. On the other hand, the opening of LTCCs is tightly controlled to prevent intracellular Ca^2+^overload. A major intrinsic negative feedback mechanism is the Ca^2+^-dependent inactivation (CDI) of the widely distributed voltage-gated Ca^2+^ channels [5], [6], [7], [8], [9], [10]. From the pioneering descriptions [11], CDI manifests as the hallmark time-dependent current decay during prolonged depolarization but also determines the voltage-dependent availability of Ca^2+^ channel during double-pulse protocols. Early studies of CDI were mainly focused on Ca^2+^ entry, but SR Ca^2+^ release also contributes significantly to the CDI [5]. In cardiac myocytes, depletion of SR Ca^2+^ stores or abolition of SR Ca^2+^ release causes a reduction of CDI [3], [12], whereas increasing SR Ca^2+^ loading results in CDI enhancement [13]. CDI depends linearly on the rate and magnitude of SR Ca^2+^ release from the RyRs [12]. Thus, ∼70% of CDI that occurs during ECC in rat ventricular myocytes arises from SR Ca^2+^ released, which might reduce Ca^2+^ influx during action potential up to 50% [14], [15]. Furthermore, it has been shown that SR Ca^2+^ release dominates CDI initially, then, as [Ca^2+^]_i_ decreases due to SR Ca^2+^ reuptake, the SR dependent contribution declines with participation from Ca^2+^ entry via *I_Ca_* dominating [16]. Now, SR Ca^2+^ release from the RyRs results in discrete and localized rises of [Ca^2+^]_i_ (Ca^2+^ sparks) triggered by *I*_Ca_ [4]. The large local releases of Ca^2+^during CICR modulate in turn LTCC [3], [12], suggesting that discrete Ca^2+^ cross-signaling occurs in the microdomains of LTCC-RyRs [17].

Over the past decades there has been rapid progress toward understanding the molecular basis for CDI, cumulating with the identification of constitutively complexed calmodulin (CaM) with the Ca^2+^channel as the specific resident Ca^2+^ sensor [5], [6], [7], [8], [9], [10]. CaM relies on its N and C lobes to detect spatiotemporal aspects of the Ca^2+^ signal for regulation of Ca^2+^ channels [6]. In cardiac myocytes, for example, C lobe–mediated CDI of LTCCs appears to be an essential regulator of action potential duration [18], whereas in nerve terminals, N lobe–mediated CDI appears to underlie use-dependent short-term plasticity of synaptic transmitter release via Ca^2+^ channels [19]. More recently, a model has been proposed where the C lobe of CaM senses local [Ca^2+^]_i_ as a result of Ca^2+^ influx from the LTCC, while the N-lobe can operate as a tunable detector of global or local [Ca^2+^]_i_ arising from distant Ca^2+^ sources [8]. In addition, an N-terminal cytoplasmic region of LTCCs has been shown to restrict CaM to respond almost exclusively to local Ca^2+^ signals in the immediate vicinity of the channel pore [7]. These results gave rise to the concept of nanodomaincontrol of the CDI. This was at first focused on Ca^2+^ influx as source for modulation of the channel, formulated upon measurements from nonexcitable cells that lack CICR nanodomains. A pertinent question is, therefore, whether and how discrete and local SR Ca^2+^release participates to the Ca^2+^ control of LTCC?

We have chosen to further explore this question by comparing RyR^R4496C^ catecholaminergic polymorphic ventricular tachycardia (CPVT) mutant mice to their wild type littermates (WT) by simultaneous recordings of *I_Ca_* and the evoked [Ca^2+^]_i_ transients and Ca^2+^ sparks. CPVT is a severe inherited cardiac disorder that manifests as malignant exercise-emotion-triggered arrhythmias leading to syncope and sudden death. Mutations in the cardiac RyR account for an autosomal-dominant form in approximately 50% of CPVT cases. In knock-in transgenic mouse model (CPVT mice), the R4496C mutation of the RyR increased the Ca^2+^ sensitivity of RyR, leading to diastolic Ca^2+^ leak and arrhythmogenic triggered activity [20]. Because the RyR Ca^2+^ leak is increased in this CPVT mouse model we investigated several key steps in the process of ECC that might provide insights into local [Ca^2+^]_i_ control of LTCC.

## Results

### Increased CICR-gain at low voltage in CPVT cells

**Figure 1:**
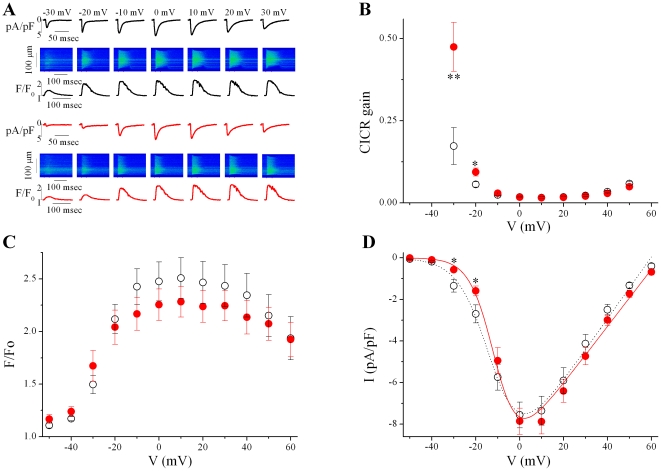
CICR gain is increased in CPVT mice due to reduced *I_Ca_* at low voltages. *A*. Representative examples of Ca^2+^ entry and release fluxes simultaneously recorded in WT (black traces) and CPVT (red traces) myocytes. Beneath the images is the corresponding profile of fluorescence, expressed as F/F_0_, where F is fluorescence and F_0_ is diastolic fluorescence, after background correction. *B*. Voltage-dependent Ca^2+^ induced-Ca^2+^ release gain (CICR-gain) decreased monotonically, giving rise to an L-shaped in CPVT (filled symbols, n = 14) and WT (open symbols, n = 16) cells. *C & D*. Voltage dependence of peak [Ca^2+^]_i_ transients (C) and peak of *I_Ca_* density (D) displayed bell-shaped, graded function with the membrane potential. * P<0.05 and ** P<0.005.

The ability of the SR to amplify the trigger Ca^2+^ influx, or CICR-gain, reflects not only the operation of the fundamental processes that underlie normal ECC, but also those involved in important pathological conditions of the heart, such as triggered arrhythmias produced by uncontrolled SR Ca^2+^ release [4], [21]. Figure 1A shows representative experiments of *I_Ca_* traces (normalized to cell capacitance) and line scan images of the evoked [Ca^2+^]_i_ transients (using the fluorescence Ca^2+^ indicator fluo-3) recorded in freshly isolated ventricular myocytes from wild type (WT, top) and CPVT (bottom) mouse hearts using simultaneous patch-clamp current recording and high resolution confocal Ca^2+^ imaging techniques [22]. The measurements were used to calculate the CICR-gain, defined as the ratio of the peak [Ca^2+^]_i_ transient (F/F_0_) over the corresponding Ca^2+^ influx through the LTCC, calculated as the *I_C_*_a_ integral, in response to voltage steps (Figure 1B) [22]. At −30 and −20 mV, the curve for CPVT cells bends upward and deviates significantly from that obtained from the WT cells, then the CICR-gain curve essentially overlaps with that of the WT. This happens even though the SR Ca^2+^ content was constant, as estimated by the integral of the caffeine-evoked inward current (in pC: 381.2±70.1 *vs *326.0±92.6, in 13 WT *vs *14CPVT cells, respectively, P>0.05) [23].

The enhanced CICR-gain at more negative voltages, despite maintained SR Ca^2+^ load, might reflect an increased efficiency of crosstalk between LTCCs and RyRs. Figures 1C and D compares the average voltage dependence of peak [Ca^2+^]_i_ transient and *I_Ca_* in WT and CPVT myocytes from experiments such as those shown in Figure 1A. Both *I_Ca_* and [Ca^2+^]_i_ transients displayed bell-shaped, graded function with the membrane potential. Whereas no difference on [Ca^2+^]_i_ transient was observed between WT and CPVT cells at any potential (Figure 1C), the peak *I_Ca_*-voltage relationships showed significant reductions at low voltages in CPVT cells, leaving unmodified the maximal *I_Ca _*(Figure 1D).

These results indicated that the RyRR4496C mutation lowers *I_Ca_* at negative voltages without global [Ca^2+^]_i_ transient alteration, resulting in enhancement of CICR-gain, consistent with the increased Ca^2+^ sensitivity of RyRs [20]. This could be explained by a modified activity of Na^+^/Ca^2+^ exchanger (NCX), which might rapidly and reversibly alter the Ca^2+^ concentration in the vicinity of the LTCCs [24]. However, the NCX currents (normalized to cell size) showed similar values in WT and CPVT myocytes (peak current density normalized by peak caffeine-evoked [Ca^2+^]_i_ transient, as evaluated by synchronous confocal images, in pA/pF: −0.88±0.09 vs −0.82±0.13, in 10 WT vs 12 CPVT cells, respectively, P>0.05).

### Changes of voltage-dependent availability of Ca^2+^ channel reduce window current

**Figure 2 pone-0020863-g002:**
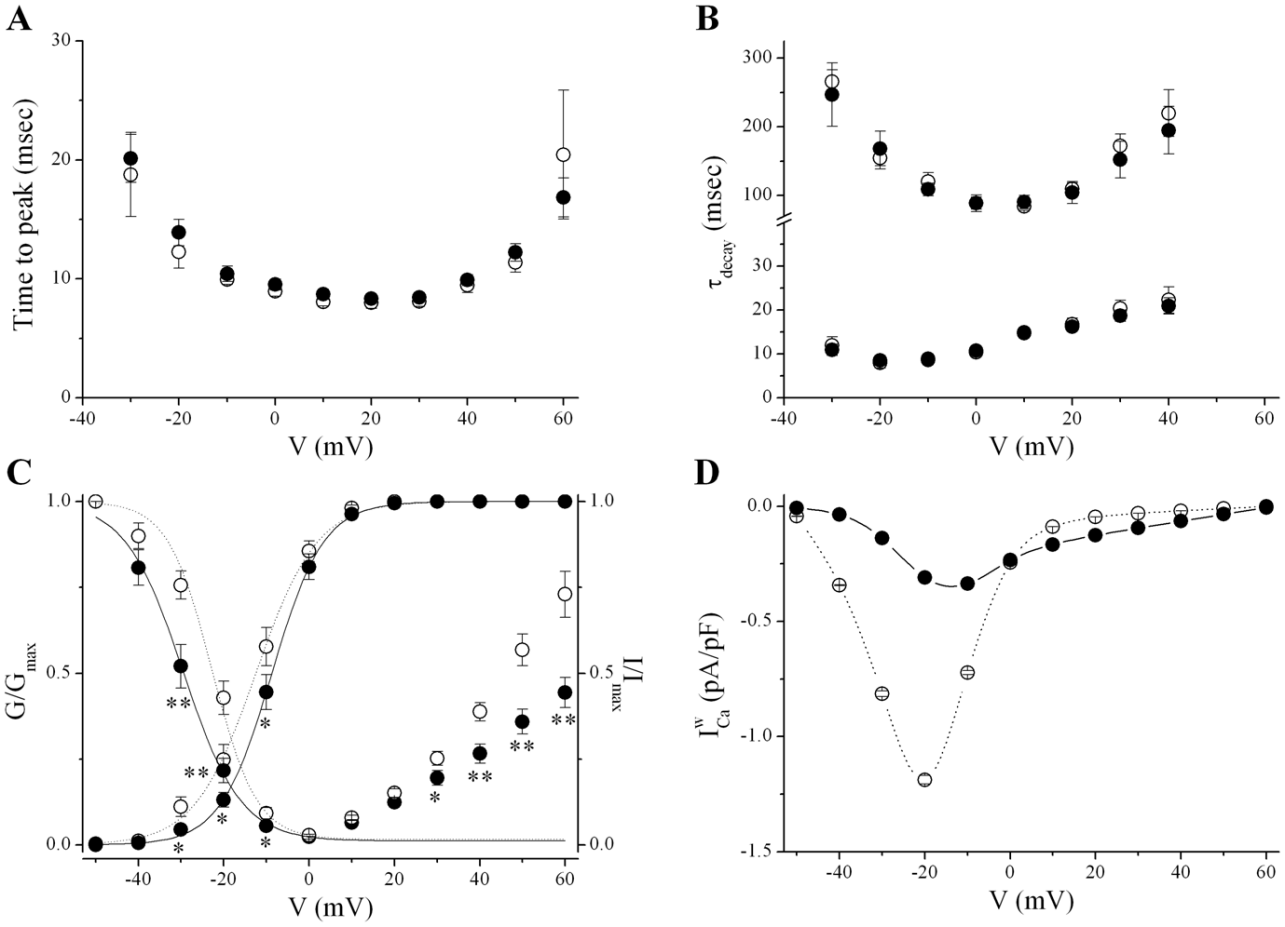
CPVT cells demonstrated rightward and leftward shifts in the voltage-dependent activation and inactivation of *I_Ca_*, respectively. *A*. Activation kinetics of *I_Ca_* over the whole voltage range were not significantly different between WT (open symbols) and CPVT (filled symbols) cells. *B*. The time course of inactivation of *I_Ca_*, which encompass slow and fast components, were similar in WT and CPVT cells at all voltages studied. *C.* Superimposed voltage-dependence of *I_Ca_* activation and inactivation. *I_Ca_* activationis shifted to more positive values in CPVT *vs* WT cells, whereas inactivation of *I_Ca_* is shifted to more hyperpolarized potential in CPVT cells compared with WT cells. *D*. Voltage dependence of *I_Ca_* window current (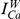) display a bell-shaped voltage-dependence, however, the peak of 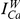 is reduced in CPVT cells (continuous line) compared to WT cells (dashed line). * P<0.05 and ** P<0.005.

A change in the time- and/or voltage-dependence of *I_Ca_* kinetics could account for the observed alteration of *I_Ca_*. Over the whole voltage range, neither activation (Figure 2A) nor inactivation (Figure 2B) kinetics of *I_Ca_* were significantly different between WT and CPVT cells. In addition, the increase in current area upon repetitive stimuli (during trains of voltage pulses), or frequency-dependent facilitation, was not modified in CPVT cells (data not shown).

Difference in the availability of *I_Ca_* as function of the voltage might underlie the reduced peak current density at low voltages. The activation-voltage relationships were constructed by converting the peak current values from each current-voltage relationship data set to the chord conductance using the equation: *G* = *I*/(*V*-*E_rev_*) and then the ratio *G/G_max_* were plotted against the membrane potential (*E_rev_* and *G_max_* as determined by the current-voltage fits [25]). In both CPVT and WT cells, the relations rise sigmoidally from 0 to 1 over the range −50 to +30 mV, but the voltage ranges for activation of *I_Ca_* in CPVT cells were significantly more positive than for WT cells (Figure 2C). To determine the activation variable (*d ∞*), curves through the data points were fitted by the Boltzmann function *d ∞ *(*V *) = 1*/*{1+exp[(*V_0.5_*-*V *)*/k*]}, where *V_0.5_* is the potential at which the conductance is half maximally activated and *k* is the slope factor describing the steepness of the curve. Whereas k was unchanged, a significant rightward shift in *V_0.5 _*was observed in CPVT cells compared to WT (Table 1).

**Table 1:** Parameters of Boltzmann fittings of activation (d∞) and inactivation (f∞) curves (in mV).

	WT				CPVT			
	d∞		f∞		d∞		f∞	
Control	V_0.5_−12.6±1.4	k6.3±0.5	V_0.5_−22.8±1.1	k6.1±0.5	V_0.5_−8.9±1.2*	k5.3±0.2	V_0.5_−29.8±1.6*	k5.9±0.5
	(n = 14)		(n = 8)		(n = 16)		(n = 8)	
Iso	−21.1±1.6#	4.7±0.3#	−36.5±1.3#	4.4±0.6#	−20.0±2.2†	4.6±0.5†	−36.3±1.4†	4.2±0.8†
	(n = 6)		(n = 6)		(n = 7)		(n = 7)	
BAPTA	−16.2±0.9#	3.6±0.3#	−24.5±0.4	5.3±0.2	−15.4±1.0†	4.0±0.2†	−24.0±0.4†	5.8±0.2
	(n = 12)		(n = 12)		(n = 13)		(n = 11)	
Thapsi	−17.5±1.7#	4.7±0.5#	−24.7±1.0	5.8±0.5	−19.9±2.1†	4.2±0.6†	−25.1±0.5†	6.5±0.3
	(n = 11)		(n = 10)		(n = 8)		(n = 8)	
Ryanod	−7.6±0.7#	5.2±0.1	−25.7±0.8#	5.2±0.1	−		−	
	(n = 12)		(n = 10)					
W7	−9.2±0.5#	5.9±0.2	−31.7±0.6#	5.5±0.1	−8.3±0.4	5.9±0.2	−31.9±0.4	5.5±0.2
	(n = 12)		(n = 11)		(n = 13)		(n = 12)	
CALP2	−8.2±1.6#	6.3±0.3	−32.9±1.2#	5.9±0.5	−7.7±1.2	5.6±0.5	−30.7±1.1	6.0±0.2
	(n = 8)		(n = 8)		(n = 11)		(n = 10)	

*p<0.05 vs WT;

# p<0.05 vs control WT;

† p<0.05 vs control CPVT.

Even if this shift, toward slightly more positive membrane potentials, might be involved in the reduction of *I_Ca_* at low voltages, maintained *I_Ca_* at more positive voltages might reflect alteration in *I_Ca_* inactivation. We then determined isochronic inactivation with a double-pulse protocol, in which the relative amplitude of *I_Ca _*during the test pulse (normalized to the maximum test current, *I_max_*) is proportional to the fraction of available channels at that given time. As shown in Figure 2C, the relations are sigmoid over −50 to 0 mV voltage range, but at prepulse potentials more positive than 0 mV the extent of inactivation decreased resulting in an U-shaped inactivation curve, consistent with inactivation arising from voltage- and Ca^2+^-dependent processes (VDI and CDI, respectively) [11]. In contrast to activation, we observed that the voltage range where channels are experiencing inactivation is significantly more negative in CPVT than in WT cells. In addition, the turn up of inactivation curve at positive potentials is significantly reduced in CPVT. Data negative to 0 mV from individual cells were fit to the Boltzmann equation *f∞(V)* = (1-*A*)/{1+exp[(*V*-*V_0.5_*)/*k*]}+*A*; where *V_0.5_* is the potential of half-maximal inactivation, *k* is the slope factor, *A* is the amplitude of the non-inactivating *I_Ca_*. A significant leftward shift is observed for *V_0.5_* in CPVT compared to WT cells (Table 1).

The overlap of activation and inactivation voltage curves delimits a window current region that allows the channels to be tonically active at these membrane potentials, as the channel population dynamically equilibrates among open, closed and inactivated states, resulting in a steady-state current [26]. Due to opposite shifts in activation and inactivation curves (Figure 2C), the inactivation-activation overlap “window area” for *I_Ca_* was substantially reduced in CPVT cells compared with controls. The Ca^2+^ window current (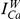) was estimated by the product of available channels (*d∞.f∞*) and the relative peak conductance using the classical Hodgkin and Huxley formulation: *G_max_*.*d*_∞_.*f*_∞_.(*V*-*E_rev_*). A marked reduction in 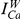 was observed in CPVT myocytes compared with WT myocytes (Figure 2D). Of note, under β-adrenergic stimulation, which induced ∼1.5 fold increase in *G_max_* (in pS/pF: 124.1±4.0 *vs* 173.9±13.1 in 6 WT cells and 134.1±7.5 *vs* 210.3±28.3 in 7 CPVT cells, before and after 1 µmol/L isoproterenol, respectively), no more differences in activation or inactivation parameters (Table 1) were observed between WT and CPVT myocytes.

### Ca^2+^ dependence of reduced window current

**Figure 3:**
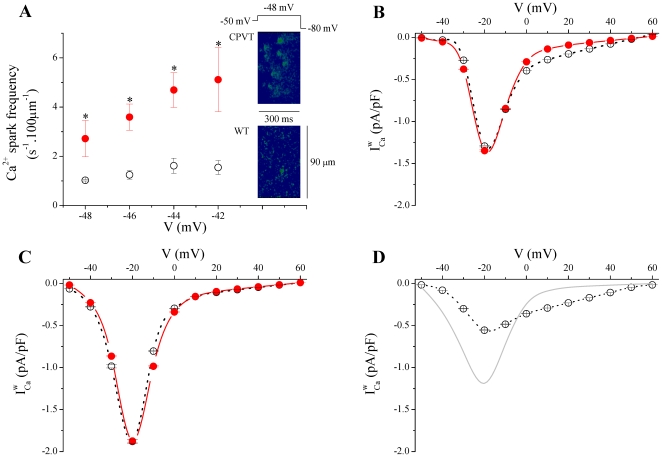
Increased Ca^2+^ spark occurrence limits *I_Ca_* window current (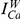). *A*. Analyze of the patch clamped-Ca^2+^ sparks revealed that the average of Ca^2+^ sparks frequencies in CPVT cells (filled circles, n = 10) were significantly higher compared to WT cells (open circles, n = 3). * P<0.05. *Right insets*. Representative examples of line-scan images of Ca^2+^ sparks elicited by depolarizing step to −48 mV. *B & C*. BAPTA dialysates (B) and thapsigargin-treatment (C) eliminates the difference in 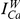 between CPVT and WT cells. *D*. In presence of Ryanodol in the perfusion solution, 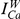 is reduced in WT cells compared to control condition, shown as light gray line.

Our observations furthered investigation on the molecular mechanism by which the R4496C mutation in RyR alters the 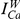. In cardiomyocytes, due to the close functional association of LTCCs with RyRs in the dyadic space, the Ca^2+^ flux through either channel modifies the activity of the other channel. Thereby SR Ca^2+^ release influences at least partly *I_Ca_* [5]. Whereas [Ca^2+^]_i_ transient is unaltered in CPVT cells (Figure 1C), CPVT myocyte show a marked increased in RyR Ca^2+^ leak, which might be visualized as Ca^2+^ sparks [4], [20]. We analyzed voltage-activated Ca^2+^ sparks in patch-clamped cells, applying small depolarizing steps from −48 to −42 mV for 300 ms, in 2 mV increments. At these voltages, the number of activated LTCCs is limited and it is possible to resolve the evoked Ca^2+^ sparks [22]. Figure 3A shows that the Ca^2+^ spark frequencies were significantly higher over the studied voltage range in CPVT compared to WT cells. As previously reported on spontaneous Ca^2+^ sparks [20], no other change in spatiotemporal spark properties were denoted in *I_Ca_*-evoked Ca^2+^ sparks (data not shown). These results lead us to probe the plausible Ca^2+^ dependence of the cross-signaling between *I_Ca_* and RyR in the reduced 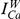. To explore this possibility, further experiments were carried out by increasing the Ca^2+^-buffering capacity of the cytoplasm through the introduction of exogenous buffer inside the patch pipette. We used the fast Ca^2+^chelator 1,2-bis (2-ethane-N,N,N′,N′-tetraacetic acid (BAPTA, 10 mmol/L). When compared to control conditions, increasing the cytoplasmic Ca^2+^-buffering capacity with BAPTA in CPVT cells completely blunted the shifts in d*∞* activation and f*∞* inactivation variables eliminating the difference with WT cells (Table 1). As a results, 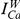 was merely identical to that observed in WT cells when [Ca^2+^]_i_ is clamped with BAPTA (Figure 3B). This suggests that local [Ca^2+^]_i_ increases lead to the reduced 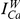 in CPVT mice. To test the contribution of the SR in the Ca^2+^-dependent effects in 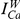, we functionally disabled intracellular Ca^2+^ stores by application of the SR Ca^2+^ ATPase inhibitor (thapsigargin, 5 µmol/L) to block the ability of the cell to pump Ca^2+^ into the SR and to deplete the store by consecutive depolarizations to 0 mV. Consequently, SR Ca^2+^ release was blunted. In the same way as for buffering [Ca^2+^]_i_, abolishing SR Ca^2+^ release restored 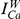 in CPVT cells to WT levels (Figure 3C) by preventing the shifts in *d∞* and *f∞* variables (Table 1). It is noteworthy that both BAPTA dialysates and thapsigargin-treatment induced in WT cells a significant shift in the hyperpolarizing direction and a steepening of the activation curves (Table 1), resulting in a slight increased peak 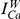 (compare Figures 1 to [Fig pone-0020863-g002] 3B and C). This emphasizes the physiological relevance of RyR Ca^2+^ leak-mediated 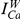 reduction.To confirm this interpretation, we exposed WT cells to 50-µmol/L Ryanodol, which has been shown to increase the overall Ca^2+^ spark rate without global change on SR Ca^2+^ load [27]. Similarly to CPVT cells, upon Ryanodol exposition, the activation of *I_Ca_* in WT cells was shifted toward positive voltages while the inactivation was shifted toward negative voltages (Table 1, Ryanod) resulting in a decrease of 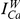 (Figure 3D).

### CaM antagonists promoted window current reduction

**Figure 4:**
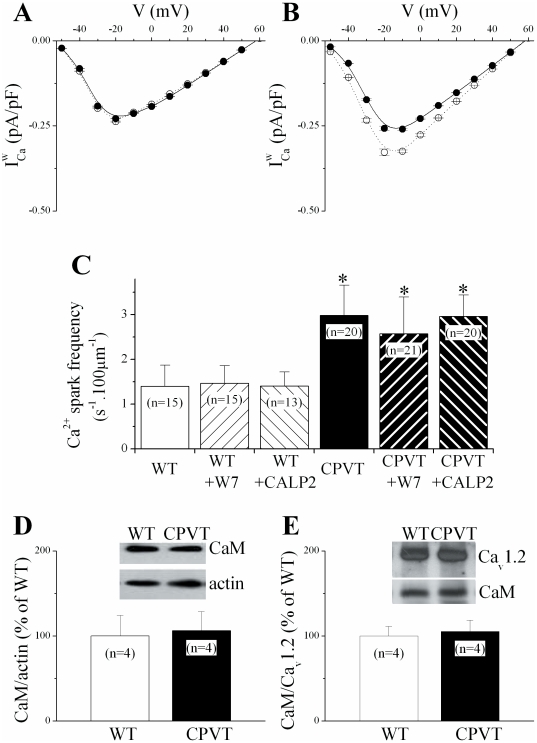
Calmodulin inhibition reduced 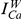 in WT cells. *A & B*. CaM antagonists, W7 (*A*) or CALP2 (*B*) reduced 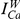 in WT cells (dashed line) without affecting it in CPVT cells (continuous line). *C*. Comparison of the Ca^2+^ sparks occurrence at rest in myocytes from WT (open bars) and CPVT (closed bars) cells in control conditions and after incubation with W7 (left hatched bars) or CALP2 (right hatched bars). * P<0.05 vs WT. *D & E*. Representative immunoblots and quantification of CaM protein levels in cardiac heart lysates (*D*, normalized to the corresponding actin level and normalized to respective controls) and detected in the membrane fraction (*E*, normalized to the corresponding Ca_v_1.2 level and normalized to respective controls) from WT (open bars, n = 4) and CPVT (closed bars, n = 4) mice.

Collectively, our findings suggest that increased RyR Ca^2+^ leak limits 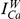. But what could the essential mechanistic ingredient of this effect be? The ubiquitous Ca^2+^ binding protein calmodulin (CaM) has a central role in determining LTCC Ca^2+^ sensitivity [5], [6], [7], . We therefore proceeded to probe the plausible involvement of CaM in reduction of 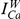. As a first approximation of events that may be CaM dependent, we used [*N*-(6-aminohexyl)-5-chloro-1-naphthalenesulfonamide] (W7, 1 µmol/L), a water-soluble, cell membrane–permeant competitive antagonist of Ca^2+^/CaM [28]. Surprisingly, 30-min W7 cell-treatment did not affect either availability variables (Table 1) or 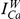 in CPVT cells (Figure 4A), whereas it shifted both activation curve to more positive voltages and inactivation curve to more negative voltages (Table 1) in WT cells compared to control conditions. Consequently, W7-treated WT cells behave such as CPVT cells, showing a reduced 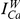 (Figure 4A). Because the results obtained with W7 should not be taken as absolute proof of a CaM-dependent process, we repeated the experiments with a cell-permeable CaM-inhibitory peptide, CALP2, designed to bind to EF-hand four-amino acid sequence of CaM [29]. Similarly to W7-treatment, a 60-min incubation with 100-µmol/L CALP2 did not affect either 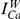 (Figure 4B) or availability variables (Table 1) in CPVT cells. However, in WT cells CALP2 significantly shifted the activation curve rightward and the inactivation curve leftward (Table 1) leading to a reduction of 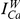 towards the level corresponding to that of CPVT cardiomyocytes (Figures 4B). As an important control, neither in CPVT nor in WT CaM antagonists altered the diastolic RyR activity in intact cells, visualized *in situ* by confocal microscopy as spontaneous Ca^2+^ sparks frequencies (Figure 4C). As well, the amount of soluble (Figure 4D) and membrane bound CaM (Figure 4E) are not altered in CPVT mice hearts compared to control WT, consistent with unaltered *I_Ca_* decay kinetics (Figure 2B).

## Discussion

The purpose of our study was to examine whether discrete and local increase of SR Ca^2+^ release has any effect on *I_Ca_* with respect to nanodomain control of LTCCs. Using CPVT mice as a model with high spontaneous RyR-generated Ca^2+^ leak, our results pinpoint that the increase in RyRCa^2+^ leak caused opposite shifts in activation and in activation voltage curves of the cardiac LTCC. The resulting reduction of window current is prevented by manoeuvres that minimize variations in [Ca^2+^]_i_ due to SR Ca^2+^ release. Surprisingly, application of CaM antagonists did not have any effect on the cells from the CPVT mouse but altered the inactivation and activation curves in the WT mouse to make these more like the CPVT mouse, revealing a new paradigm for CaM-dependent effect on LTCC gating. These effects might represent an adaptive mechanism to cytotoxic SR Ca^2+^ leak and Ca^2+^ overload.

### RyRCa^2+^ leak limits
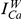


Analysis of skeletal myotubes derived from RyR-knockout (dyspedic) mice has revealed that in addition to the orthograde signal of ECC, there is also a retrograde signal whereby RyR promotes the Ca^2+^ conducting activity of the skeletal LTCC [30]. But this bi-directional coupling is thought to result from direct physical protein-protein interaction [30]. Nevertheless, it has been recently suggested that this feedback mechanism, which results in reduction of 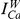, might also depend on local SR Ca^2+^ signaling [2]. Despite some controversial results, no such structural interaction has been found in cardiac cells. However, the close physical association between LTCCs and RyRs that in cardiac muscle takes place at the narrow site of the tubulo-reticular junction where each LTCC is closely associated with a group of RyRs [4], creates a bi-directional Ca^2+^ signaling microdomain such that Ca^2+^ flux through one channel modifies the functional behavior of the other channel. In this study, we show that either buffering intracellular Ca^2+^ or eliminating SR Ca^2+^ release erased the difference in 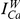 between CPVT and WT mice. Thus, we conclude that the mechanism underlying the reduced 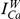 in CPVT mice is SR Ca^2+^ dependent. In cardiac cells, SR Ca^2+^ release is indeed the major component determining CDI [3], [12], [13], [14], [15], [16]. However, these studies as well as the majority of CDI analyses, relied almost exclusively on the accelerated current inactivation during depolarizing voltage steps whereas the CDI manifestation on current availability [11] has not been yet the subject of in depth investigation. Nonetheless, when compared to neonatal cardiomyocytes, where there is poor T-tubule development, adult cardiomyocytes show a reduced 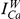 [31] and a slight reduction in *I_Ca_* availability has been observed with static elevation of [Ca^2+^]_i_ [32].

Although a global increase in [Ca^2+^]_i_ can participate to CDI, there is evidence suggesting that local Ca^2+^ signaling between RyRs and LTCCs might mediate a Ca^2+^ functional crosstalk between LTCCs and RyRs [3], [16], [17]. Changes in local subsarcolemmal Ca^2+^ caused by the alteration of normal Ca^2+^ extrusion via NCX could be involved. But we observed no difference in NCX activity between WT and CPVT cells. Such an alteration in Ca^2+^ driving force, as well as in surface charges, will be difficult to reconcile with the absence of modification in peak *I_Ca_* density, apparent reversal potential and opposite shifts of activation and inactivation voltage ranges. Long term changes are known to complicate the mechanistic attribution of cause and effect in genetically modified animals. One might thus suggest that the observed enhancement of inactivation of LTCCs could reflect a survival adaptation to increased [Ca^2+^]_i_, rather than a mechanistic insight into the functional coupling between LTCCs and RyRs. However, acute intervention buffering Ca^2+^ (Figure 3B) or depleting the SR Ca^2+^ store (Figure 3C) restores 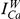 in CPVT cells to WT levels by preventing the shifts in *d∞* and *f∞* variables. These considerations let us suggest a tonic effect rather than long term adaptation. The alteration in Ca^2+^ signaling observed in CPVT cells is the increase in RyR Ca^2+^ leak. Indeed, the *I_Ca_* evoked [Ca^2+^]_i_ transients (Figure 1) were not altered. This is consistent with our previous observations and the normal cardiac function described under basal conditions in the CPVT mice, whereas a rate-dependent defect exists [20]. The increased RyR Ca^2+^ leak seems then to be insufficient to precipitate by itself changes in [Ca^2+^]_i_ transient under basal stimulation condition, effect which might reflect a sufficient time interval to maintain the physiological SR Ca^2+^ load, as we observed by caffeine application. Then, we conclude that the tonic reduction in 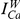 is mediated through local changes of [Ca^2+^]_i_ in the restricted space where LTCCs and RyRs are located in the dyadic junctions, a mechanism paralleling the local and global [Ca^2+^]_i_ sensing for the CDI of LTCCs [6], [7], [8]. This interpretation is further supported by the use of Ryanodol, which mimics RyR Ca^2+^ leak [27], although we cannot exclude a direct effect of this ryanoid on LTCCs.

### A new paradigm of CaM effect on LTCC gating

Our results indicate that RyR Ca^2+^ leak influence gating properties of the LTCCs. One intriguing but remarkable aspect of our findings is that the changes in the voltage-dependence of LTCC activation and inactivation were not paralleled by changes in the kinetic properties of *I_Ca_* (Figure 2A & B), similar to that observed by others [33]. Whereas this might indicate that recovery from the inactivated state was impeded, this interpretation will be difficult to reconcile with the maintenance of *I_Ca_* peak density at more depolarized voltages (Figure 1D). This emphases the complex nature of *I_Ca_* inactivation mechanisms, and thus might not reflect the same process. The absence of influences on *I_Ca_* decay suggested that discrete Ca^2+^ control is unlikely on LTTC open state, whereas shifted steady-state inactivation suggested a stronger effect on inactivated state than on rested state. However, the conformational distinct Ca^2+^ channel populations (resting, open and inactivated states) are closely interrelated. We reasoned on the possible crosstalk between the CDI and VDI gating of the LTCCs, such that a change in the stability of the open state is predicted to affect both VDI and CDI gating [34]. In this way, we suspected that the RyR Ca^2+^ leak induced enhancement of inactivation might induce the rightward shift in the activation. In fact, conformational changes caused by Ca^2+^ binding to the resident CaM, the primary initiatory event for CDI [5], [6], [7], [8], may allosterically reduce activation gating [10] and functionally link the VDI machinery [9], [35]. We observed that CaM antagonists had no effect on CPVT but reduced 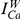 in WT. These results echo the shift in *I_Ca_* availability curve to more negative potential with calmidozolium, another CaM antagonist [36] and are consistent with previous results in recombinant systems. Similarly to our results, splice variants or mutants of the LTCC IQ motif for CaM binding show a rightward shift in activation and a leftward shift in inactivation [37], [38], whereas CaM overexpression shifts the activation to more negative potential [39]. This prompt us to suggest that the RyR Ca^2+^ leak-dependent reduced 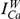 inversely related to CaM is due to an intrinsic effect on LTCC. Besides its role as a signal transduction molecule, CaM also functions as a ubiquitous endogenous fast Ca^2+^ buffer, an effect frequently overlooked [40]. We therefore propose that Ca^2+^ buffer capacity of CaM normally prevents Ca^2+^ to access to other regulator sites, since free CaM is locally enriched in the vicinity of the channels [41]. CaM antagonists in WT cells or increased RyR Ca^2+^ leak in CPVT cells outdo this CaM effect, allowing Ca^2+^ feedback to other Ca^2+^ sensor domains involved in CDI [7], [24], [35].

### Pathophysiological perspectives

Taken together, our study identifies a new Ca^2+^ regulatory mechanism acting as a powerful switch that determines LTCC gating by interfering with CaM modulation. This effect might serve as a compensatory response to counteract excessive SR Ca^2+^ release and SR store overload [42], and thus participates to the SR Ca^2+^ load decrease observed at high stimulation frequencies [20]. One might speculate on a yin-yang effect of the diastolic Ca^2+^ leakage on trigger activities for fatal acquired or genetic cardiac arrhythmias and sudden death. Elevation of diastolic [Ca^2+^]_i_ through increased Ca^2+^ spark frequency is regarded as an arrhythmogenic mechanism: Ca^2+^ sparks are believed to participate as crucial events in the initiation and propagation of Ca^2+^ waves in cardiomyocytes and the elimination of cytosolic Ca^2+^ via the NCX generates a depolarizing current, which can give rise to delayed after depolarizations (DADs) [4]. Conversely, the 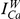 has a central role in arrhythmogenesis in the setting of action potential (AP) prolongation because it can reverse repolarization to create fluctuation of membrane voltage during the repolarization phase of the AP, or early after depolarizations (EADs) [26]. EADs may give rise to salvos of premature APs leading to after contractions through a subsequent secondary SR Ca^2+^ release, triggered activity and promote reentrant arrhythmias such as Torsades de pointes [43]. Thus, diastolic Ca^2+^ leak would promote DADs [21], while would be protective from EADs by reducing 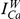. Our results indeed echo a prevention of EAD induced Torsades de pointes by W7 through a decrease of 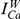 [33], [44].

## Methods

Experiments were performed on male and female heterozygous RyR2^R4497C^ mice (CPVT) and their WT littermates (F3 to F5 generation), as previously described [20], [22], in accordance to the ethical principles laid down by the French (Ministry of Agriculture) and ECC directive 96/609/EEC and was approved by the *Comité Régional d' Ethique sur l'expérimentation animale* of *Languedoc-Roussillon* on the Use and Care of Animals. All persons who participated in the experiments had the training and authorization to do so (authorization B34-172-16 for animal facility manager).

### Mice

The generation of RyR^R4496C^ knock-in mice was previously described [45]. Mice were age-matched littermates (between 4 to 6 months old and weighing 22–25 g) maintained on a C57BL/6 background after >3 backcrosses to C57BL/6.

### Cell isolation

Isolated ventricular myocytes from knock-in mouse-model carrier of the RyR R4496C mutation (CPVT) and their gender-matched littermates wild-type (WT) were prepared using an enzymatic perfusion method. Animals were treated with heparin (1000 units kg^-1^) and anaesthetized with Na^+^ pentobarbital (50 mg/kg) administered intraperitoneally. The heart was excised rapidly via a thoracotomy and the pericardium was removed. The heart was placed in ice-cold (0 °C) oxygenated Tyrode solution containing (mmol/L): NaCl 130, NaH_2_PO_4_ 0.4, NaHCO_3_ 5.8, MgCl_2_ 0.5, KCl 5.4, glucose 22, Hepes 25 and insulin 10^−3^ (titrated to pH 7.4 with NaOH). The aorta was cannulated above the aortic valve and was perfused by gravity (70 cm column height) with warm (37 °C), preoxygenated Tyrode solution supplemented with 0.1 mmol/L EGTA for 2 min. Enzyme solution containing 1 g/L collagenase Type II (Worthington) in Tyrode solution supplemented with 0.1 mmol/L CaCl_2_ was then perfused until the aortic valve was digested (attested by the increased outflow of perfusate). The heart was transferred to a Petri dish containing enzyme solution supplemented with 2 g/L bovine serum albumin (BSA) and gently shaken for 2–3 min at 37 °C to disperse individual myocytes. The resulting cell suspension was filtered through a 250 µm nylon mesh and centrifuged for 3 min at 20 g. The cell pellet was suspended in Tyrode solution supplemented with 0.5 mmol/L CaCl_2_ and 2 g/L BSA and was centrifuged again at the same speed. Finally, the cell pellet was suspended in storage solution comprising Tyrode solution supplemented with 1 mmol/L CaCl_2_ and 2 g/L BSA.

### Patch-clamp and fluorescence measurements

Whole-cell currents were monitored with an Axopatch 200A patch-clamp amplifier. Capacitance compensation was optimized and series resistance was compensated by 40–80%. Ca^2+^ images were simultaneously acquired with a confocal microscope (MetaZeiss LSM510, objective oil immersion x40, numerical aperture 1.2) in line-scan mode (1.5 msec/line). *I_Ca_* and fluorescence signals were simultaneously digitized (Digidata 1200, Axon Instruments) and acquired at sampling rate of 100 µsec using pClamp 8.1. During experiments, cells were superfused with an external solution containing (in mmol/L) 140 NaCl, 0.5 MgCl_2_, 5 CsCl, 1.8 CaCl_2_, 5.5 glucose, 5 Hepes (pH 7.4), while the patch pipette was filled with a solution containing (in mmol/L) 130 CsCl, 1 MgCl_2_, 1 NaH_2_PO_4_, 3.6 Na_2_ phosphocreatine, 5 MgATP, 10 HEPES and 0.05 Fluo-3 pentapotassium salt (Molecular Probes); pH 7.2. *I_Ca_* and SR Ca^2+^ releases were elicited by 100 msec voltage steps in 10 mV increments from −50 to +60 mV, every 10 seconds. Prior to it, to allow steady-state SR load a voltage protocol including 4 voltage steps (150 msec) from −80 to 0 mV was apply and voltage-gated Na^+^ channels were inactivated by a 500-msec ramp from −80 to −42 mV [22].

The activation kinetic of *I_Ca_* was measured for every depolarizing step as the time from the onset of the voltage step to the peak of current.

The time course of inactivation of *I_Ca_* was determined by analysis of the decay phase of current traces in response to voltage steps. Best fits were obtained with an equation including a sum of two exponentials plus a constant expressed as *A*_fast_exp(-*t*/*τ_fast_*)+*A*_slow_exp(-*t*/*τ_slow_*)+*A*_0_, where τ and *A* are the time constant and the initial amplitude of the two components subscripted fast and slow, respectively, and *A*_0_ is the amplitude of the time-independent component.

Isochronic inactivation were performed with a double pulse protocol, in which a conditioning prepulse of variable amplitude (in the −50 to +60 mV range, from −80 mV) and 250 msec in duration (long enough to produce complete inactivation at each potential) was followed by a test pulse to 0 mV (selected based on the voltage at which peak *I_Ca_* was maximum) and 100 msec in duration.

Fluo-3 fluorescence was excited with the 488-nm line of an argon ion laser. Emitted fluorescence was measured at wavelengths over 515 nm. Image acquisition was made in the line-scan mode. A single myocyte was scanned repetitively along a line parallel to the longitudinal cell axis. Image processing and analysis were performed using IDL software (Research Systems), as previously described [22], [23]. Briefly, each image was background-subtracted. The fluorescence transient was obtained by averaging the fluorescence values in a 1.4-µm frame over time. Amplitude was measured as the maximum value of F/F_0_, where F is the fluorescence signal and F_0_ is the basal fluorescence (measured as the average of the 50 lowest values on the fluorescence transient).

For SR Ca^2+^ load estimation, myocytes were previously stimulated (4 voltage 150 msec steps from −80 to 0 mV) for 1 min, then 10 mmol/L caffeine was added, the fluorescence image was recorded by confocal microscopy and the associated NCX current was recorded by patch clampand integrated to estimate the amount of Ca^2+^ released by the SR [23].

Spontaneous Ca^2+^ sparks (Figure 3A) were imaged on intact Fluo-3AM loaded cells superfused with Tyrode solution (in mmol/L: NaCl 140, MgCl_2_ 1.1, CaCl_2_ 1.8, KCl 4, glucose 10 and Hepes 10; pH 7.4).

#### Drugs

BAPTA and W7 were purchased from Calbiochem; CALP2 and Ryanodine were from Tocris Bioscience; and other chemical products were from Sigma. Ryanodol was generated as previously described [27].

#### Western blotting

Mouse heart homogenates were prepared with homogenization buffer (in mmol/L: sucrose 300, sodium-fluoride 20, HEPES 20, Aprotinin 5.2 10^−4^, Benzamidine 10^−2^, Leupeptin, 12 10^∼3^; PMSF 0.1, 0.5% sodium desoxicholate, 0.1% SDS, pH 7.2) using a Potter-Elvehjem and spun at 2,000 *g* for 10 min. Membrane fractions were isolated by ultracentrifugation at 40 000 *g* for 30 minutes at 4°C [21]. Protein concentration was assessed by the Bradford method. 25 µg of protein were fractionated on gradient (8 to 20%) SDS-PAGE gels, transferred onto PVDF membranes (30 min in the semidry transfer chamber at 15 V using the 0.45 µm Amersham Hybond-P membrane, GE Healthcare Biosciences, Waukesha, WI, USA) and probed with anti-calmodulin monoclonal antibody (1∶500, Thermo Scientific, Waltham, MA, USA) or ant-Ca_V_1.2 policlonal antibody (1∶1000, Millipore Co, Billerica, MA, USA), in TBS buffer (in mmol/L: Tris-HCl 50, NaCl 150, +0.1% v/v Tween 20, pH 7.4) with 1% w/v non-fat dried milk powder. Membranes were blocked overnight with 5% w/v non-fat dried milk powder in TBS buffer before primary antibody addition. Membranes were incubated 1 h with corresponding secondary peroxidase-conjugate goat antiserum (diluted 1∶5,000 in TBS, EMD Chemicals Inc, Gibbstown, NJ, USA). Signals were developed by chemiluminiscence (Supersignal West Pico Chemiluminescent substrate, Thermo Scientific, Waltham, MA, USA). The relative amount of protein on the blots was determined by densitometry using KodakID Software (v. 3.635, Molecular Imaging Systems, New Haven CT, USA). Actin signals were detected in the same blots with anti-actin serum (1∶20000, Sigma-Aldrich, Inc. México) and used as loading controls.

#### Statistical Analysis

Data are presented as means ± SEM and compared using t test or an unequal variance t statistic (Welch test), when appropriated, with the PAST program (http://folk.uio.no/ohammer/past/). Differences with values of P<0.05 were considered significant.

## References

[1] Berridge MJ, Bootman MD, Roderick HL (2003). Calcium signalling: dynamics, homeostasis and remodelling.. Nat Rev Mol Cell Biol.

[2] Andronache Z, Hamilton SL, Dirksen RT, Melzer W (2009). A retrograde signal from RyR1 alters DHP receptor inactivation and limits window Ca2+ release in muscle fibers of Y522S RyR1 knock-in mice.. Proc Natl Acad Sci U S A.

[3] Sham JS, Cleemann L, Morad M (1995). Functional coupling of Ca2+ channels and ryanodine receptors in cardiac myocytes.. Proc Natl Acad Sci U S A.

[4] Cheng H, Lederer WJ (2008). Calcium sparks.. Physiol Rev.

[5] Budde T, Meuth S, Pape HC (2002). Calcium-dependent inactivation of neuronal calcium channels.. Nat Rev Neurosci.

[6] Liang H, DeMaria CD, Erickson MG, Mori MX, Alseikhan BA (2003). Unified mechanisms of Ca2+ regulation across the Ca2+ channel family.. Neuron.

[7] Dick IE, Tadross MR, Liang H, Tay LH, Yang W (2008). A modular switch for spatial Ca2+ selectivity in the calmodulin regulation of CaV channels.. Nature.

[8] Tadross MR, Dick IE, Yue DT (2008). Mechanism of local and global Ca2+ sensing by calmodulin in complex with a Ca2+ channel.. Cell.

[9] Findeisen F, Minor DL (2009). Disruption of the IS6-AID linker affects voltage-gated calcium channel inactivation and facilitation.. J Gen Physiol.

[10] Tadross MR, Ben Johny M, Yue DT (2010). Molecular endpoints of Ca2+/calmodulin- and voltage-dependent inactivation of Ca(v)1.3 channels.. J Gen Physiol.

[11] Brehm P, Eckert R (1978). Calcium entry leads to inactivation of calcium channel in Paramecium.. Science.

[12] Adachi-Akahane S, Cleemann L, Morad M (1996). Cross-signaling between L-type Ca2+ channels and ryanodine receptors in rat ventricular myocytes.. J Gen Physiol.

[13] Sipido KR, Callewaert G, Carmeliet E (1995). Inhibition and rapid recovery of Ca2+ current during Ca2+ release from sarcoplasmic reticulum in guinea pig ventricular myocytes.. Circ Res.

[14] Grantham CJ, Cannell MB (1996). Ca2+ influx during the cardiac action potential in guinea pig ventricular myocytes.. Circ Res.

[15] Puglisi JL, Yuan W, Bassani JW, Bers DM (1999). Ca(2+) influx through Ca(2+) channels in rabbit ventricular myocytes during action potential clamp: influence of temperature.. Circ Res.

[16] Sun H, Leblanc N, Nattel S (1997). Mechanisms of inactivation of L-type calcium channels in human atrial myocytes.. Am J Physiol.

[17] Sham JS (1997). Ca2+ release-induced inactivation of Ca2+ current in rat ventricular myocytes: evidence for local Ca2+ signalling.. J Physiol.

[18] Alseikhan BA, DeMaria CD, Colecraft HM, Yue DT (2002). Engineered calmodulins reveal the unexpected eminence of Ca2+ channel inactivation in controlling heart excitation.. Proc Natl Acad Sci U S A.

[19] Xu J, Wu LG (2005). The decrease in the presynaptic calcium current is a major cause of short-term depression at a calyx-type synapse.. Neuron.

[20] Fernandez-Velasco M, Rueda A, Rizzi N, Benitah JP, Colombi B (2009). Increased Ca2+ sensitivity of the ryanodine receptor mutant RyR2R4496C underlies catecholaminergic polymorphic ventricular tachycardia.. Circ Res.

[21] Gomez AM, Rueda A, Sainte-Marie Y, Pereira L, Zissimopoulos S (2009). Mineralocorticoid modulation of cardiac ryanodine receptor activity is associated with downregulation of FK506-binding proteins.. Circulation.

[22] Gomez AM, Valdivia HH, Cheng H, Lederer MR, Santana LF (1997). Defective excitation-contraction coupling in experimental cardiac hypertrophy and heart failure.. Science.

[23] Pereira L, Matthes J, Schuster I, Valdivia HH, Herzig S (2006). Mechanisms of [Ca2+]i transient decrease in cardiomyopathy of db/db type 2 diabetic mice.. Diabetes.

[24] Isaev D, Solt K, Gurtovaya O, Reeves JP, Shirokov R (2004). Modulation of the voltage sensor of L-type Ca2+ channels by intracellular Ca2+.. J Gen Physiol.

[25] Benitah JP, Vassort G (1999). Aldosterone upregulates Ca(2+) current in adult rat cardiomyocytes.. Circ Res.

[26] Hirano Y, Moscucci A, January CT (1992). Direct measurement of L-type Ca2+ window current in heart cells.. Circ Res.

[27] Ramos-Franco J, Gomez AM, Nani A, Liu Y, Copello JA (2010). Ryanodol action on calcium sparks in ventricular myocytes.. Pflugers Arch.

[28] Hidaka H, Kobayashi R (1992). Pharmacology of protein kinase inhibitors.. Annu Rev Pharmacol Toxicol.

[29] Villain M, Jackson PL, Manion MK, Dong WJ, Su Z (2000). De novo design of peptides targeted to the EF hands of calmodulin.. J Biol Chem.

[30] Dirksen RT (2002). Bi-directional coupling between dihydropyridine receptors and ryanodine receptors.. Front Biosci.

[31] Cohen NM, Lederer WJ (1988). Changes in the calcium current of rat heart ventricular myocytes during development.. J Physiol.

[32] Altamirano J, Bers DM (2007). Effect of intracellular Ca2+ and action potential duration on L-type Ca2+ channel inactivation and recovery from inactivation in rabbit cardiac myocytes.. Am J Physiol Heart Circ Physiol.

[33] Qi X, Yeh YH, Chartier D, Xiao L, Tsuji Y (2009). The calcium/calmodulin/kinase system and arrhythmogenic afterdepolarizations in bradycardia-related acquired long-QT syndrome.. Circ Arrhythm Electrophysiol.

[34] Yue DT, Backx PH, Imredy JP (1990). Calcium-sensitive inactivation in the gating of single calcium channels.. Science.

[35] Peterson BZ, Lee JS, Mulle JG, Wang Y, de Leon M (2000). Critical determinants of Ca(2+)-dependent inactivation within an EF-hand motif of L-type Ca(2+) channels.. Biophys J.

[36] Klockner U, Isenberg G (1987). Calmodulin antagonists depress calcium and potassium currents in ventricular and vascular myocytes.. Am J Physiol.

[37] Soldatov NM, Oz M, O'Brien KA, Abernethy DR, Morad M (1998). Molecular determinants of L-type Ca2+ channel inactivation. Segment exchange analysis of the carboxyl-terminal cytoplasmic motif encoded by exons 40-42 of the human alpha1C subunit gene.. J Biol Chem.

[38] Zuhlke RD, Reuter H (1998). Ca2+-sensitive inactivation of L-type Ca2+ channels depends on multiple cytoplasmic amino acid sequences of the alpha1C subunit.. Proc Natl Acad Sci U S A.

[39] Ravindran A, Lao QZ, Harry JB, Abrahimi P, Kobrinsky E (2008). Calmodulin-dependent gating of Ca(v)1.2 calcium channels in the absence of Ca(v)beta subunits.. Proc Natl Acad Sci U S A.

[40] Kubota Y, Putkey JA, Shouval HZ, Waxham MN (2008). IQ-motif proteins influence intracellular free Ca2+ in hippocampal neurons through their interactions with calmodulin.. J Neurophysiol.

[41] Mori MX, Erickson MG, Yue DT (2004). Functional stoichiometry and local enrichment of calmodulin interacting with Ca2+ channels.. Science.

[42] Fabiato A (1985). Simulated calcium current can both cause calcium loading in and trigger calcium release from the sarcoplasmic reticulum of a skinned canine cardiac Purkinje cell.. J Gen Physiol.

[43] Tomaselli GF, Zipes DP (2004). What causes sudden death in heart failure?. Circ Res.

[44] Mazur A, Roden DM, Anderson ME (1999). Systemic administration of calmodulin antagonist W-7 or protein kinase A inhibitor H-8 prevents torsade de pointes in rabbits.. Circulation.

[45] Cerrone M, Colombi B, Santoro M, di Barletta MR, Scelsi M (2005). Bidirectional ventricular tachycardia and fibrillation elicited in a knock-in mouse model carrier of a mutation in the cardiac ryanodine receptor.. Circ Res.

